# 2-Hy­droxy-*N*′-[2-(6-meth­oxy­naphthalen-2-yl)propano­yl]benzohydrazide

**DOI:** 10.1107/S1600536812012731

**Published:** 2012-03-31

**Authors:** Shaaban Kamel Mohamed, Peter N. Horton, Mehmet Akkurt, Mustafa R. Albayati, Herman Potgeiter

**Affiliations:** a Chemistry and Environmental Division, Manchester Metropolitan University, Manchester M1 5GD, England; b School of Chemistry, University of Southampton, Highfield, Southampton SO17 1BJ, England; c Department of Physics, Faculty of Sciences, Erciyes University, 38039 Kayseri, Turkey; d School of Research, Enterprise & Innovation, Manchester Metropolitan University, Manchester M1 5GD, England

## Abstract

In the title compound, C_21_H_20_N_2_O_4_, the naphthalene ring system makes a dihedral angle of 84.5 (3)° with the benzene ring, and the –C(=O)–N(H)–N(H)–C(=O)– torsion angle is 70.7 (7)°, so that the mol­ecule is twisted. An *S*(6) ring motif is formed *via* an intra­molecular O—H⋯O hydrogen bond. In the crystal, mol­ecules are linked by N—H⋯O and C–H⋯O hydrogen bonds into supra­molecular layers in the *ab* plane.

## Related literature

For the pharmaceutical applications of naproxen [systematic name: (+)-6-meth­oxy-α-methyl-2-naphthalene acetic acid], see: Teplyakov *et al.* (1993 ▶); Bozdag *et al.* (2001 ▶). For the synthesis of potential biologically active compounds based on the structure of naproxen, see: Sharma *et al.* (2003 ▶); Kumar *et al.* (2010 ▶). For related structures, see: Yathirajan *et al.* (2007 ▶); Liang *et al.* (2008 ▶). For hydrogen-bond motifs, see: Etter *et al.* (1990 ▶).
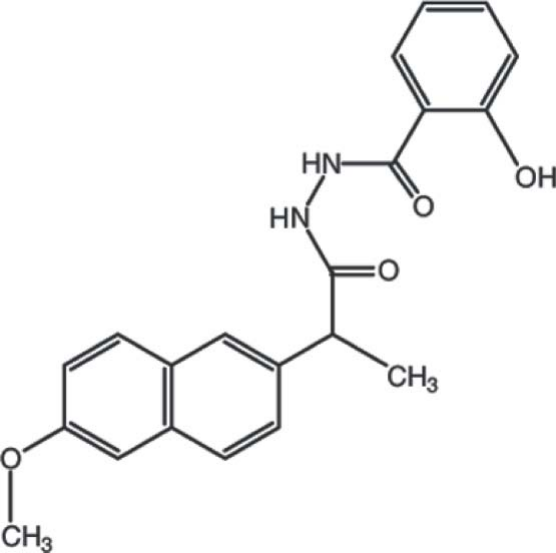


## Experimental

### 

#### Crystal data

C_21_H_20_N_2_O_4_
*M* *_r_* = 364.39
Orthorhombic, 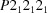

*a* = 4.851 (6) Å
*b* = 10.407 (12) Å
*c* = 36.65 (4) Å
*V* = 1850 (4) Å^3^
*Z* = 4
Mo *K*α radiation
μ = 0.09 mm^−1^
*T* = 100 K
0.14 × 0.05 × 0.01 mm

#### Data collection

Rigaku Saturn724+ diffractometer
Absorption correction: multi-scan (*CrystalClear-SM Expert*; Rigaku, 2011 ▶) *T*_min_ = 0.987, *T*_max_ = 0.999
5963 measured reflections
2303 independent reflections
1169 reflections with *I* > 2σ(*I*)
*R*_int_ = 0.102

#### Refinement

*R*[*F* ^2^ > 2σ(*F* ^2^)] = 0.084
*wR*(*F* ^2^) = 0.189
*S* = 1.09
2303 reflections
257 parameters
3 restraints
H atoms treated by a mixture of independent and constrained refinement
Δρ_max_ = 0.30 e Å^−3^
Δρ_min_ = −0.24 e Å^−3^

### 

Data collection: *CrystalClear-SM Expert* (Rigaku, 2011 ▶); cell refinement: *CrystalClear-SM Expert*; data reduction: *CrystalClear-SM Expert*; program(s) used to solve structure: *SIR97* (Altomare *et al.*, 1999 ▶); program(s) used to refine structure: *SHELXL97* (Sheldrick, 2008 ▶); molecular graphics: *ORTEP-3 for Windows* (Farrugia, 1997 ▶) and *PLATON* (Spek, 2009 ▶); software used to prepare material for publication: *WinGX* (Farrugia, 1999 ▶) and *PLATON*.

## Supplementary Material

Crystal structure: contains datablock(s) global, I. DOI: 10.1107/S1600536812012731/tk5071sup1.cif

Structure factors: contains datablock(s) I. DOI: 10.1107/S1600536812012731/tk5071Isup2.hkl

Supplementary material file. DOI: 10.1107/S1600536812012731/tk5071Isup3.cml

Additional supplementary materials:  crystallographic information; 3D view; checkCIF report

## Figures and Tables

**Table 1:** Hydrogen-bond geometry (Å, °)

*D*—H⋯*A*	*D*—H	H⋯*A*	*D*⋯*A*	*D*—H⋯*A*
N1—H1*N*⋯O2^i^	0.87 (3)	2.19 (3)	2.974 (8)	149 (5)
N2—H2*N*⋯O4^ii^	0.86 (5)	2.31 (5)	3.072 (8)	149 (5)
O4—H4⋯O3	0.82 (5)	1.83 (5)	2.618 (7)	161 (6)
C12—H12⋯O2^i^	0.98	2.36	3.274 (9)	155
C21—H21⋯O3^iii^	0.93	2.54	3.465 (9)	174

Symmetry codes: (i) 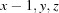; (ii) 
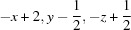
; (iii) 
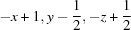
.

**Fig. 1.**
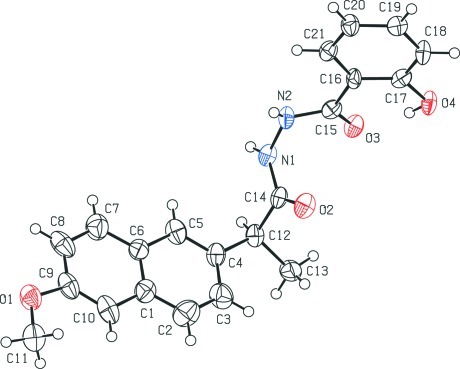
The molecular structure of (I), showing the labelling of the non-H atoms and displacement ellipsoids drawn at the 50% probability level.

**Fig. 2.**
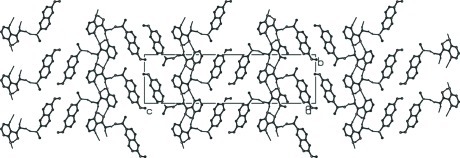
View of the packing and hydrogen bonding (dashed lines) of (I) down the a axis, in the unit-cell. H atoms not involved in hydrogen bonds have been omitted for clarity.

## supplementary crystallographic information

### Comment

A commonly prescribed non-steroidal anti-inflammatory drug (NSAID) is naproxen [(+)-6-methoxy-α-methyl-2-naphthalene acetic acid] (Teplyakov *et al.*, 1993; Bozdag *et al.*, 2001). It is used in the treatment of painful and inflammatory conditions like rheumatoid arthritis, spondilytis, and osteoarthritis (Sharma *et al.*, 2003; Kumar *et al.*, 2010). As part of our study on the functionalization of naproxen moiety, we report the structure of the title compound, (I), with the aim of synthesising potential biologically active compounds based on the core structure. of naproxen

In the title compound (I), (Fig. 1), the N1—N2 bond length of 1.427 (8) Å, indicates a single bond. All bond lengths in (I) are within normal ranges (Yathirajan *et al.*, 2007; Liang *et al.*, 2008). The naphthalene ring (C1—C10) is planar, with a maximum deviation of -0.007 (7) Å for the C4 atom. This ring makes a dihedral angle of 84.5 (3)° with the hydroxybenzene ring. The —C14(═O)—N1(H)—N2(H)—C15(═O)— torsion angle is 70.7 (7) °.

An intramolecular O—H···O hydrogen bond which generates an S(6) ring motif (Etter *et al.*, 1990) is observed in the molecular structure (Table 1). The crystal structure is stabilized by intermolecular N—H···O and C—H···O hydrogen bonds (Table 1, Fig. 2) which connect molecules into supramolecular layers in the *ab* plane.

### Experimental

A mixture of 0.01 mol of 2-(6-methoxy-2-naphthyl)propanoyl chloride and 0.01 mol 2-hydroxybenzohydrazide in tetrahydrofuran was heated at 339 K for three hours. The reaction progress was monitored by TLC until completed. The mixture was then poured on cold water to afford the solid product which was filtered off, dried and recrystallized from ethanol in 72% yield with *M*.pt: at 482–484 K. Suitable crystals for X-ray diffraction were obtained by slow evaporation of a diluted ethanolic solution of the product over two days.

### Refinement

The hydroxyl- and amide-H atoms were located in difference density maps, and were refined with the (O, N)—H distance restraints of 0.82 (2) Å and 0.86 (2) Å, respectively, and with free *U*_iso_. The remaining H atoms were positioned geometrically and refined using a riding model with C—H = 0.93, 0.96 and 0.98 Å for aromatic-, methyl- and methine-H, respectively, with *U*_iso_(H) = 1.2 or 1.5 *U*_eq_(C).

The crystal used was very small (0.01 x 0.05 x 0.14) but was the best available after repeated recrystallizations. As such a reasonable number of the higher angle diffractions were indistinguishable from the background noise. The crystal was exposed to the X-rays for as long as required until reached a level where increasing the exposure did not result in the observation of further high angle data. From the reflections collected the structure could be readily determined, such that hydrogen atom positions could be seen in the difference map, even though most were later fixed using standard riding positions (the OH and NH hydrogen atoms were allowed some freedom of position whilst restraining their distances to 0.82 Å and 0.86 Å, respectively). In the absence of significant anomalous scattering, 1052 Friedel pairs were averaged. One poorly fitted reflection (0 1 1) was omitted from the refinement.

### Crystal data

**Table:**

C_21_H_20_N_2_O_4_	*F*(000) = 768
*M**_r_* = 364.39	*D*_x_ = 1.308 Mg m^−^^3^
Orthorhombic, *P*2_1_2_1_2_1_	Mo *K*α radiation, λ = 0.71073 Å
Hall symbol: P 2ac 2ab	Cell parameters from 4928 reflections
*a* = 4.851 (6) Å	θ = 2.0–30.1°
*b* = 10.407 (12) Å	µ = 0.09 mm^−^^1^
*c* = 36.65 (4) Å	*T* = 100 K
*V* = 1850 (4) Å^3^	Sheet, colourless
*Z* = 4	0.14 × 0.05 × 0.01 mm

### Data collection

**Table:**

Rigaku Saturn724+ diffractometer	2303 independent reflections
Radiation source: Rotating Anode	1169 reflections with *I* > 2σ(*I*)
Confocal monochromator	*R*_int_ = 0.102
Detector resolution: 28.5714 pixels mm^-1^	θ_max_ = 27.3°, θ_min_ = 2.6°
profile data from ω–scans	*h* = −6→4
Absorption correction: multi-scan (*CrystalClear-SM Expert*; Rigaku, 2011)	*k* = −13→12
*T*_min_ = 0.987, *T*_max_ = 0.999	*l* = −47→23
5963 measured reflections	

### Refinement

**Table:**

Refinement on *F*^2^	Secondary atom site location: difference Fourier map
Least-squares matrix: full	Hydrogen site location: inferred from neighbouring sites
*R*[*F*^2^ > 2σ(*F*^2^)] = 0.084	H atoms treated by a mixture of independent and constrained refinement
*wR*(*F*^2^) = 0.189	*w* = 1/[σ^2^(*F*_o_^2^) + (0.0587*P*)^2^] where *P* = (*F*_o_^2^ + 2*F*_c_^2^)/3
*S* = 1.09	(Δ/σ)_max_ < 0.001
2303 reflections	Δρ_max_ = 0.30 e Å^−^^3^
257 parameters	Δρ_min_ = −0.24 e Å^−^^3^
3 restraints	Extinction correction: *SHELXL97* (Sheldrick, 2008), FC^*^=KFC[1+0.001XFC^2^Λ^3^/SIN(2Θ)]^-1/4^
Primary atom site location: structure-invariant direct methods	Extinction coefficient: 0.008 (2)

### Special details

**Table:**

Geometry. Bond distances, angles *etc*. have been calculated using the rounded fractional coordinates. All su's are estimated from the variances of the (full) variance-covariance matrix. The cell e.s.d.'s are taken into account in the estimation of distances, angles and torsion angles
Refinement. Refinement on *F*^2^ for ALL reflections except those flagged by the user for potential systematic errors. Weighted *R*-factors *wR* and all goodnesses of fit *S* are based on *F*^2^, conventional *R*-factors *R* are based on *F*, with *F* set to zero for negative *F*^2^. The observed criterion of *F*^2^ > σ(*F*^2^) is used only for calculating -*R*-factor-obs *etc*. and is not relevant to the choice of reflections for refinement. *R*-factors based on *F*^2^ are statistically about twice as large as those based on *F*, and *R*-factors based on ALL data will be even larger.

### Fractional atomic coordinates and isotropic or equivalent isotropic displacement parameters (Å^2^)

**Table:**

	*x*	*y*	*z*	*U*_iso_*/*U*_eq_	
O1	0.5719 (10)	−0.0932 (4)	0.02322 (12)	0.0513 (17)	
O2	0.8625 (10)	0.5761 (4)	0.16863 (13)	0.0427 (17)	
O3	0.5850 (8)	0.7217 (4)	0.23922 (11)	0.0353 (16)	
O4	0.9808 (11)	0.8058 (4)	0.28151 (13)	0.0387 (16)	
N1	0.4392 (11)	0.5286 (5)	0.19267 (15)	0.0303 (17)	
N2	0.5549 (11)	0.5038 (5)	0.22776 (15)	0.0337 (17)	
C1	0.6354 (15)	0.2465 (7)	0.05874 (18)	0.044 (3)	
C2	0.7799 (17)	0.3603 (8)	0.0541 (2)	0.063 (3)	
C3	0.7265 (17)	0.4677 (7)	0.07585 (19)	0.055 (3)	
C4	0.5185 (14)	0.4648 (6)	0.10359 (18)	0.038 (3)	
C5	0.3727 (14)	0.3541 (6)	0.10912 (18)	0.043 (3)	
C6	0.4279 (15)	0.2393 (6)	0.08636 (17)	0.040 (3)	
C7	0.2788 (17)	0.1242 (7)	0.09106 (19)	0.056 (3)	
C8	0.3346 (17)	0.0178 (7)	0.06947 (19)	0.055 (3)	
C9	0.5405 (17)	0.0239 (7)	0.04166 (19)	0.048 (3)	
C10	0.6932 (17)	0.1321 (7)	0.03550 (19)	0.056 (3)	
C11	0.7870 (15)	−0.1005 (7)	−0.00403 (19)	0.058 (3)	
C12	0.4624 (13)	0.5834 (6)	0.12777 (16)	0.034 (2)	
C13	0.5530 (16)	0.7139 (6)	0.11123 (16)	0.046 (3)	
C14	0.6096 (15)	0.5647 (6)	0.16510 (18)	0.032 (2)	
C15	0.6505 (12)	0.6079 (6)	0.24718 (17)	0.032 (2)	
C16	0.8385 (13)	0.5788 (6)	0.27894 (16)	0.0300 (19)	
C17	0.9991 (13)	0.6797 (6)	0.29347 (17)	0.032 (2)	
C18	1.1903 (13)	0.6545 (6)	0.32135 (17)	0.036 (2)	
C19	1.2169 (14)	0.5287 (6)	0.33541 (16)	0.035 (2)	
C20	1.0521 (13)	0.4295 (6)	0.32161 (17)	0.037 (2)	
C21	0.8645 (14)	0.4527 (6)	0.29390 (16)	0.033 (2)	
H1N	0.260 (5)	0.521 (6)	0.1924 (14)	0.027 (18)*	
H2	0.91480	0.36530	0.03610	0.0750*	
H2N	0.636 (12)	0.431 (4)	0.2295 (18)	0.06 (3)*	
H3	0.82790	0.54250	0.07230	0.0660*	
H4	0.851 (9)	0.797 (6)	0.2673 (14)	0.05 (3)*	
H5	0.23850	0.35090	0.12720	0.0520*	
H7	0.14190	0.11960	0.10880	0.0670*	
H8	0.23720	−0.05810	0.07310	0.0660*	
H10	0.82820	0.13420	0.01750	0.0660*	
H11A	0.78710	−0.18460	−0.01480	0.0870*	
H11B	0.96250	−0.08480	0.00720	0.0870*	
H11C	0.75440	−0.03720	−0.02260	0.0870*	
H12	0.26350	0.58760	0.13230	0.0410*	
H13A	0.50790	0.78200	0.12790	0.0700*	
H13B	0.45870	0.72750	0.08850	0.0700*	
H13C	0.74830	0.71300	0.10710	0.0700*	
H18	1.29920	0.72050	0.33050	0.0430*	
H19	1.34370	0.51170	0.35380	0.0420*	
H20	1.06880	0.34700	0.33120	0.0440*	
H21	0.75540	0.38620	0.28500	0.0390*	

### Atomic displacement parameters (Å^2^)

**Table:**

	*U*^11^	*U*^22^	*U*^33^	*U*^12^	*U*^13^	*U*^23^
O1	0.062 (3)	0.038 (3)	0.054 (3)	0.003 (3)	−0.002 (3)	−0.010 (2)
O2	0.022 (3)	0.046 (3)	0.060 (3)	0.000 (2)	−0.006 (2)	0.003 (3)
O3	0.034 (3)	0.022 (2)	0.050 (3)	0.006 (2)	−0.002 (2)	0.004 (2)
O4	0.043 (3)	0.021 (2)	0.052 (3)	−0.006 (2)	−0.008 (3)	−0.003 (2)
N1	0.016 (3)	0.029 (3)	0.046 (3)	0.003 (3)	−0.003 (3)	0.001 (3)
N2	0.032 (3)	0.026 (3)	0.043 (3)	−0.004 (3)	−0.011 (3)	−0.001 (3)
C1	0.034 (4)	0.048 (5)	0.049 (4)	−0.002 (4)	−0.010 (4)	0.004 (4)
C2	0.050 (5)	0.064 (6)	0.074 (6)	0.001 (5)	0.011 (5)	0.002 (5)
C3	0.054 (5)	0.052 (5)	0.058 (5)	0.005 (5)	0.009 (5)	−0.016 (4)
C4	0.037 (5)	0.027 (4)	0.049 (4)	0.004 (4)	−0.004 (4)	−0.004 (3)
C5	0.044 (5)	0.035 (4)	0.051 (4)	0.011 (4)	−0.007 (4)	−0.008 (3)
C6	0.043 (5)	0.041 (4)	0.037 (4)	−0.005 (4)	−0.015 (3)	0.009 (3)
C7	0.063 (6)	0.045 (5)	0.060 (5)	−0.002 (5)	−0.006 (5)	−0.003 (4)
C8	0.076 (6)	0.046 (5)	0.044 (4)	0.021 (5)	−0.021 (5)	−0.009 (4)
C9	0.057 (6)	0.039 (4)	0.049 (4)	0.012 (5)	−0.016 (4)	−0.013 (4)
C10	0.061 (5)	0.050 (5)	0.056 (5)	0.009 (5)	−0.015 (4)	−0.019 (4)
C11	0.058 (5)	0.051 (5)	0.065 (5)	−0.002 (5)	−0.005 (5)	−0.008 (4)
C12	0.024 (4)	0.034 (4)	0.045 (4)	0.002 (3)	−0.004 (3)	−0.005 (3)
C13	0.064 (5)	0.038 (4)	0.037 (4)	−0.001 (4)	−0.008 (4)	0.005 (3)
C14	0.038 (5)	0.015 (3)	0.044 (4)	0.001 (3)	−0.001 (4)	0.000 (3)
C15	0.024 (4)	0.033 (4)	0.038 (4)	0.003 (4)	0.006 (3)	0.002 (3)
C16	0.031 (4)	0.021 (3)	0.038 (3)	−0.002 (3)	0.006 (3)	−0.011 (3)
C17	0.034 (4)	0.021 (3)	0.041 (4)	0.007 (3)	0.014 (3)	0.001 (3)
C18	0.033 (4)	0.024 (4)	0.051 (4)	−0.004 (3)	−0.010 (4)	−0.005 (3)
C19	0.031 (4)	0.038 (4)	0.036 (4)	0.002 (4)	−0.001 (3)	0.002 (3)
C20	0.041 (4)	0.027 (4)	0.042 (4)	0.001 (4)	−0.004 (4)	−0.003 (3)
C21	0.033 (4)	0.024 (4)	0.041 (4)	0.001 (3)	0.003 (3)	0.000 (3)

### Geometric parameters (Å, º)

**Table:**

O1—C9	1.402 (9)	C15—C16	1.509 (9)
O1—C11	1.446 (9)	C16—C21	1.428 (9)
O2—C14	1.239 (9)	C16—C17	1.412 (9)
O3—C15	1.260 (8)	C17—C18	1.405 (9)
O4—C17	1.386 (8)	C18—C19	1.413 (9)
O4—H4	0.82 (5)	C19—C20	1.400 (9)
N1—N2	1.427 (8)	C20—C21	1.385 (9)
N1—C14	1.359 (9)	C2—H2	0.9300
N2—C15	1.377 (8)	C3—H3	0.9300
N1—H1N	0.87 (3)	C5—H5	0.9300
N2—H2N	0.86 (5)	C7—H7	0.9300
C1—C10	1.491 (10)	C8—H8	0.9300
C1—C2	1.387 (11)	C10—H10	0.9300
C1—C6	1.430 (10)	C11—H11A	0.9600
C2—C3	1.397 (11)	C11—H11B	0.9600
C3—C4	1.433 (10)	C11—H11C	0.9600
C4—C5	1.367 (9)	C12—H12	0.9800
C4—C12	1.544 (9)	C13—H13A	0.9600
C5—C6	1.482 (9)	C13—H13B	0.9600
C6—C7	1.410 (10)	C13—H13C	0.9600
C7—C8	1.388 (10)	C18—H18	0.9300
C8—C9	1.429 (11)	C19—H19	0.9300
C9—C10	1.367 (11)	C20—H20	0.9300
C12—C13	1.551 (9)	C21—H21	0.9300
C12—C14	1.556 (9)		
			
C9—O1—C11	117.2 (5)	O4—C17—C16	123.3 (6)
C17—O4—H4	98 (4)	C17—C18—C19	119.9 (6)
N2—N1—C14	118.8 (5)	C18—C19—C20	120.0 (6)
N1—N2—C15	117.1 (5)	C19—C20—C21	120.8 (6)
C14—N1—H1N	129 (4)	C16—C21—C20	120.0 (6)
N2—N1—H1N	113 (3)	C1—C2—H2	119.00
N1—N2—H2N	114 (4)	C3—C2—H2	119.00
C15—N2—H2N	120 (4)	C2—C3—H3	119.00
C2—C1—C6	119.2 (6)	C4—C3—H3	119.00
C2—C1—C10	121.1 (7)	C4—C5—H5	120.00
C6—C1—C10	119.7 (6)	C6—C5—H5	120.00
C1—C2—C3	121.3 (7)	C6—C7—H7	120.00
C2—C3—C4	121.2 (7)	C8—C7—H7	120.00
C3—C4—C5	119.2 (6)	C7—C8—H8	120.00
C3—C4—C12	121.0 (6)	C9—C8—H8	120.00
C5—C4—C12	119.8 (6)	C1—C10—H10	121.00
C4—C5—C6	120.2 (6)	C9—C10—H10	121.00
C5—C6—C7	121.6 (6)	O1—C11—H11A	109.00
C1—C6—C7	119.5 (6)	O1—C11—H11B	110.00
C1—C6—C5	118.9 (6)	O1—C11—H11C	110.00
C6—C7—C8	120.6 (7)	H11A—C11—H11B	109.00
C7—C8—C9	120.5 (7)	H11A—C11—H11C	110.00
O1—C9—C8	112.4 (6)	H11B—C11—H11C	109.00
C8—C9—C10	122.2 (7)	C4—C12—H12	108.00
O1—C9—C10	125.3 (7)	C13—C12—H12	108.00
C1—C10—C9	117.5 (7)	C14—C12—H12	108.00
C13—C12—C14	108.9 (5)	C12—C13—H13A	109.00
C4—C12—C13	115.2 (5)	C12—C13—H13B	109.00
C4—C12—C14	108.9 (5)	C12—C13—H13C	109.00
O2—C14—N1	123.5 (6)	H13A—C13—H13B	110.00
O2—C14—C12	122.3 (6)	H13A—C13—H13C	109.00
N1—C14—C12	114.2 (6)	H13B—C13—H13C	110.00
O3—C15—N2	122.3 (5)	C17—C18—H18	120.00
N2—C15—C16	116.4 (5)	C19—C18—H18	120.00
O3—C15—C16	121.3 (5)	C18—C19—H19	120.00
C15—C16—C21	122.3 (5)	C20—C19—H19	120.00
C17—C16—C21	119.3 (6)	C19—C20—H20	120.00
C15—C16—C17	118.4 (5)	C21—C20—H20	120.00
O4—C17—C18	116.7 (5)	C16—C21—H21	120.00
C16—C17—C18	120.0 (6)	C20—C21—H21	120.00
			
C11—O1—C9—C8	−176.3 (6)	C5—C6—C7—C8	−179.9 (7)
C11—O1—C9—C10	1.3 (10)	C1—C6—C7—C8	0.9 (11)
N2—N1—C14—O2	0.8 (9)	C6—C7—C8—C9	−1.2 (11)
C14—N1—N2—C15	70.7 (7)	C7—C8—C9—O1	178.8 (7)
N2—N1—C14—C12	177.5 (5)	C7—C8—C9—C10	1.2 (12)
N1—N2—C15—C16	−164.0 (5)	O1—C9—C10—C1	−178.1 (6)
N1—N2—C15—O3	16.3 (8)	C8—C9—C10—C1	−0.8 (11)
C10—C1—C6—C7	−0.5 (10)	C4—C12—C14—N1	−102.1 (6)
C6—C1—C2—C3	0.0 (11)	C13—C12—C14—O2	−51.7 (8)
C2—C1—C6—C5	0.3 (10)	C13—C12—C14—N1	131.6 (6)
C2—C1—C10—C9	−179.6 (7)	C4—C12—C14—O2	74.7 (7)
C6—C1—C10—C9	0.5 (10)	O3—C15—C16—C17	−17.0 (9)
C10—C1—C2—C3	−179.9 (7)	O3—C15—C16—C21	164.9 (6)
C2—C1—C6—C7	179.6 (7)	N2—C15—C16—C17	163.3 (6)
C10—C1—C6—C5	−179.8 (6)	N2—C15—C16—C21	−14.9 (9)
C1—C2—C3—C4	−0.6 (12)	C15—C16—C17—O4	4.0 (9)
C2—C3—C4—C12	179.4 (7)	C15—C16—C17—C18	−175.6 (6)
C2—C3—C4—C5	0.9 (11)	C21—C16—C17—O4	−177.8 (6)
C5—C4—C12—C14	78.8 (7)	C21—C16—C17—C18	2.6 (9)
C3—C4—C12—C14	−99.7 (7)	C15—C16—C21—C20	176.1 (6)
C12—C4—C5—C6	−179.2 (6)	C17—C16—C21—C20	−2.0 (9)
C3—C4—C12—C13	22.9 (9)	O4—C17—C18—C19	178.8 (6)
C5—C4—C12—C13	−158.6 (6)	C16—C17—C18—C19	−1.5 (9)
C3—C4—C5—C6	−0.7 (10)	C17—C18—C19—C20	−0.2 (9)
C4—C5—C6—C7	−179.2 (7)	C18—C19—C20—C21	0.8 (9)
C4—C5—C6—C1	0.1 (10)	C19—C20—C21—C16	0.3 (9)

### Hydrogen-bond geometry (Å, º)

**Table:**

*D*—H···*A*	*D*—H	H···*A*	*D*···*A*	*D*—H···*A*
N1—H1*N*···O2^i^	0.87 (3)	2.19 (3)	2.974 (8)	149 (5)
N2—H2*N*···O4^ii^	0.86 (5)	2.31 (5)	3.072 (8)	149 (5)
O4—H4···O3	0.82 (5)	1.83 (5)	2.618 (7)	161 (6)
C12—H12···O2^i^	0.98	2.36	3.274 (9)	155
C21—H21···O3^iii^	0.93	2.54	3.465 (9)	174

Symmetry codes: (i) *x*−1, *y*, *z*; (ii) −*x*+2, *y*−1/2, −*z*+1/2; (iii) −*x*+1, *y*−1/2, −*z*+1/2.
